# Metabolite-Pool Networks and Biological–Non-Enzymatic Synergy in Volatile Flavor Formation by Lactic Acid Bacteria in Fermented Foods: A Review

**DOI:** 10.3390/foods15183275

**Published:** 2026-09-16

**Authors:** Hailin Tong, Jingxin Li, Yanqiu Jing, Binqiang Tian, Dongsheng Luo

**Affiliations:** Flavors and Fragrance Engineering & Technology Research Center of Henan Province, Henan Agricultural University, Zhengzhou 450051, China

**Keywords:** lactic acid bacteria, volatile flavor compounds, fermented foods, metabolic pool, co-fermentation, biological–non-enzymatic synergy

## Abstract

Volatile flavor compounds (VFCs) are major determinants of sensory quality in fermented foods. In lactic acid bacteria (LAB)-containing systems, their formation reflects precursor metabolism, strain-specific metabolic capacity, environmental and community regulation, and non-enzymatic chemistry. This review develops an integrated precursor–pool–flux framework for LAB-associated VFC formation. Carbohydrate/citrate-, protein/amino-acid-, and lipid-related routes provide the principal precursor inputs, which intersect to different extents at three functional metabolite pools centered on α-keto acids, acetyl-CoA-related metabolites, and C4–C5 TCA intermediates. These pools are treated as operational convergence and redistribution nodes rather than new biochemical classifications. Genetic capacity, intracellular regulation, cultivation conditions, and interspecies interactions modify precursor supply and flux partitioning, thereby altering quantitative VFC output. Biological–non-enzymatic coupling is considered where processing changes precursor accessibility, fermentation-derived substrates undergo subsequent chemical transformation, or biological and chemical reactions proceed concurrently. Comprehensive volatile profiling defines the resulting chemical phenotype, whereas molecular sensory and flavoromics identify aroma-active endpoints. This framework provides a mechanistic basis for interpreting LAB-associated VFC formation and guiding strain and process selection toward targeted aroma regulation.

## 1. Introduction

Lactic acid bacteria (LAB) are major starter and adjunct microorganisms in fermented-food production, where their metabolic activities contribute directly or indirectly to aldehydes, ketones, alcohols, organic acids, esters, sulfur-containing volatiles, and other matrix-dependent VFCs [1,2]. The concentration of a VFC does not necessarily predict its sensory importance because odor threshold, phase partitioning, and mixture interactions influence perceptual contribution [3,4]. Previous reviews have described LAB biochemical routes and precursor metabolism [1,5], microbial succession and key flavor compounds [2], lipid metabolism in fermented meat [6,7], microbiota interactions [8], branched-chain-amino-acid-derived aroma compounds [9], LAB metabolism in fermented vegetables [10], volatile diversity in plant fermentations [11], and analytical approaches for aroma interpretation [3]. Existing studies have established most individual components of LAB-associated flavor formation, but integration across metabolic pathways, strain variation, environmental control, microbial communities, and processing chemistry remains incomplete. Carbohydrate/citrate-, amino-acid-, and lipid-related pathways are commonly described separately, although their carbon and nitrogen fluxes converge at pyruvate, amino-acid-derived α-keto acids [12,13], acetyl-CoA-related metabolites [14,15], oxaloacetate, and α-ketoglutarate [16].

The α-keto-acid, acetyl-CoA-related, and C4–C5 TCA-intermediate pools provide operational network modules for recurrent convergence and redistribution between substrate-specific metabolism and downstream VFC formation. Each pool can receive carbon or nitrogen from more than one precursor source, and its intermediates may be partitioned among competing branches according to strain capacity, cofactor and redox state, substrate availability, and community context. Pyruvate functions both as a central carbohydrate-derived intermediate and as an α-keto acid. α-Ketoglutarate is assigned to the C4–C5 TCA-intermediate module because its principal roles here are TCA-related metabolism and amino-group acceptance. Citrate remains an upstream C6 substrate that supplies oxaloacetate and pyruvate rather than a member of the C4–C5 pool. Biological VFC formation can be resolved into three mechanistic levels. Upstream metabolism supplies precursors, shared metabolite pools define recurrent convergence and redistribution nodes, and genetic, intracellular, environmental, and community-level factors regulate precursor entry and branch-specific flux. This distinction is particularly important in natural and mixed fermentations, where yeasts, staphylococci/micrococci, molds, acetic acid bacteria, endogenous food enzymes, and processing conditions can provide precursors or catalyze conversions that LAB alone perform weakly or not at all [8,17,18].

Non-enzymatic chemistry also contributes to VFC formation, although its importance varies among food matrices and processing stages. Chemical reactions may act on fermentation-derived or fermentation-released substrates, such as α-acetolactate, organic acids, alcohols, amino acids, reducing sugars, free fatty acids, and sulfur precursors [18,19]. Their rates can also respond to fermentation-dependent changes in pH, oxidation–reduction potential (ORP), and oxygen availability [20,21,22]. When precursor dependence or physicochemical control has not been resolved experimentally, co-occurrence of microbial metabolism and chemical conversion does not establish a causal link. Biological and non-enzymatic contributions are considered according to the evidence available for each matrix and processing stage, without assuming that one process necessarily depends on the other. Mechanistic analysis begins with precursor formation, convergence at shared metabolic nodes, and regulation of branch flux. Biological and non-enzymatic routes are then examined according to whether physicochemical modification occurs before microbial conversion, chemical conversion acts on fermentation-derived substrates, or the two processes overlap in time.

Volatile profiling and molecular sensory approaches provide the chemical phenotype associated with these mechanisms. Odor-activity analysis and flavoromics are used when interpretation extends from VFC formation to characteristic aroma. Direct LAB-derived formation is assigned only when strain-, enzyme-, or controlled-conversion evidence resolves the source; otherwise, volatile changes in mixed systems are treated as LAB-associated system-level outcomes.

**Figure 1:**
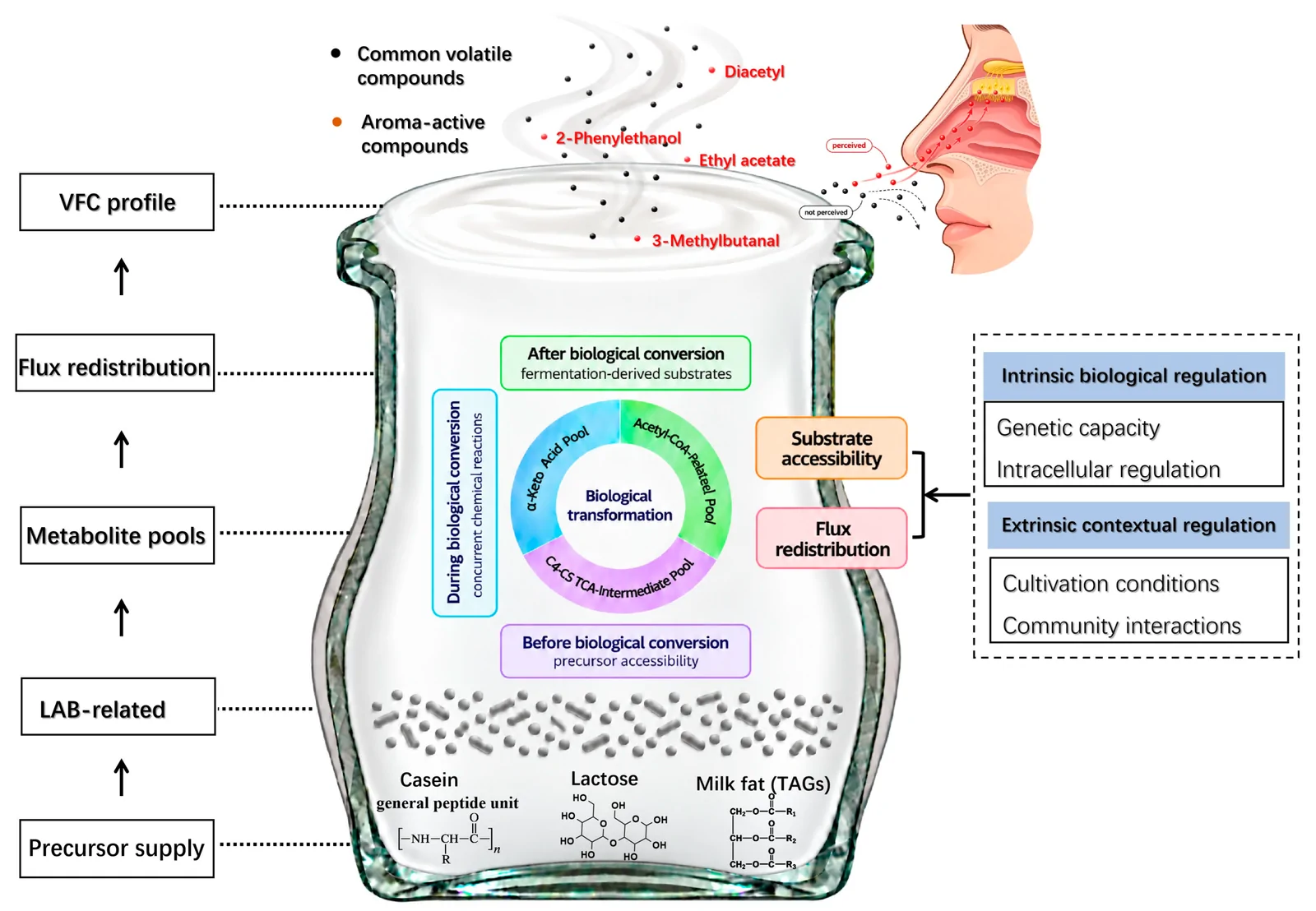
Integrated framework linking precursor metabolism to volatile flavor and key aroma formation in LAB-associated fermentation. Note. Fermented milk is shown as a representative LAB-associated food matrix for visualization; the mechanistic framework is intended to apply across fermented-food systems.

Accordingly, this review focuses on how precursor supply is connected to shared metabolic nodes, how flux is redistributed among competing branches, how biological and non-enzymatic reactions jointly shape the resulting volatile profile, and which of these products ultimately contribute to characteristic aroma. By integrating metabolic organization, environmental and community control, chemical transformation, volatile phenotypes, and sensory relevance within a single mechanistic structure, the review aims to explain why particular strains or process interventions produce specific VFC responses. Its broader significance lies in identifying experimentally accessible control points that can support rational LAB strain selection and process optimization, thereby advancing fermented-food flavor research from end-point volatile comparisons toward mechanism-based regulation of aroma-active outcomes, as shown in Figure 1.

### Literature Search and Selection

To improve transparency, relevant literature for this narrative, mechanism-oriented review was identified through Web of Science, Scopus, PubMed/MEDLINE, ScienceDirect, SpringerLink, Wiley Online Library, and Google Scholar, with reference tracking of key reviews and mechanistically relevant primary studies. Search terms covered lactic acid bacteria, volatile flavor compounds, aroma, flavoromics, sensomics, molecular sensory science, GC–olfactometry, odor activity value, aroma recombination, omission test, metabolite network, metabolite pool, pyruvate metabolism, citrate metabolism, α-keto acids, acetyl-CoA, amino acid catabolism, lipid metabolism, co-fermentation, microbial interactions, non-enzymatic reactions, Maillard reaction, and Strecker degradation.

Priority was given to studies reporting strain identity, food matrix, precursor conversion, volatile quantification, and sensory or odor-activity relevance. Genomic, transcriptomic, or correlative omics studies without direct functional validation were used primarily to support mechanistic hypotheses rather than causal attribution. As a narrative review, formal PRISMA-style screening counts were not compiled.

## 2. Biological Formation of Flavor Precursors and VFCs

**Figure 2:**
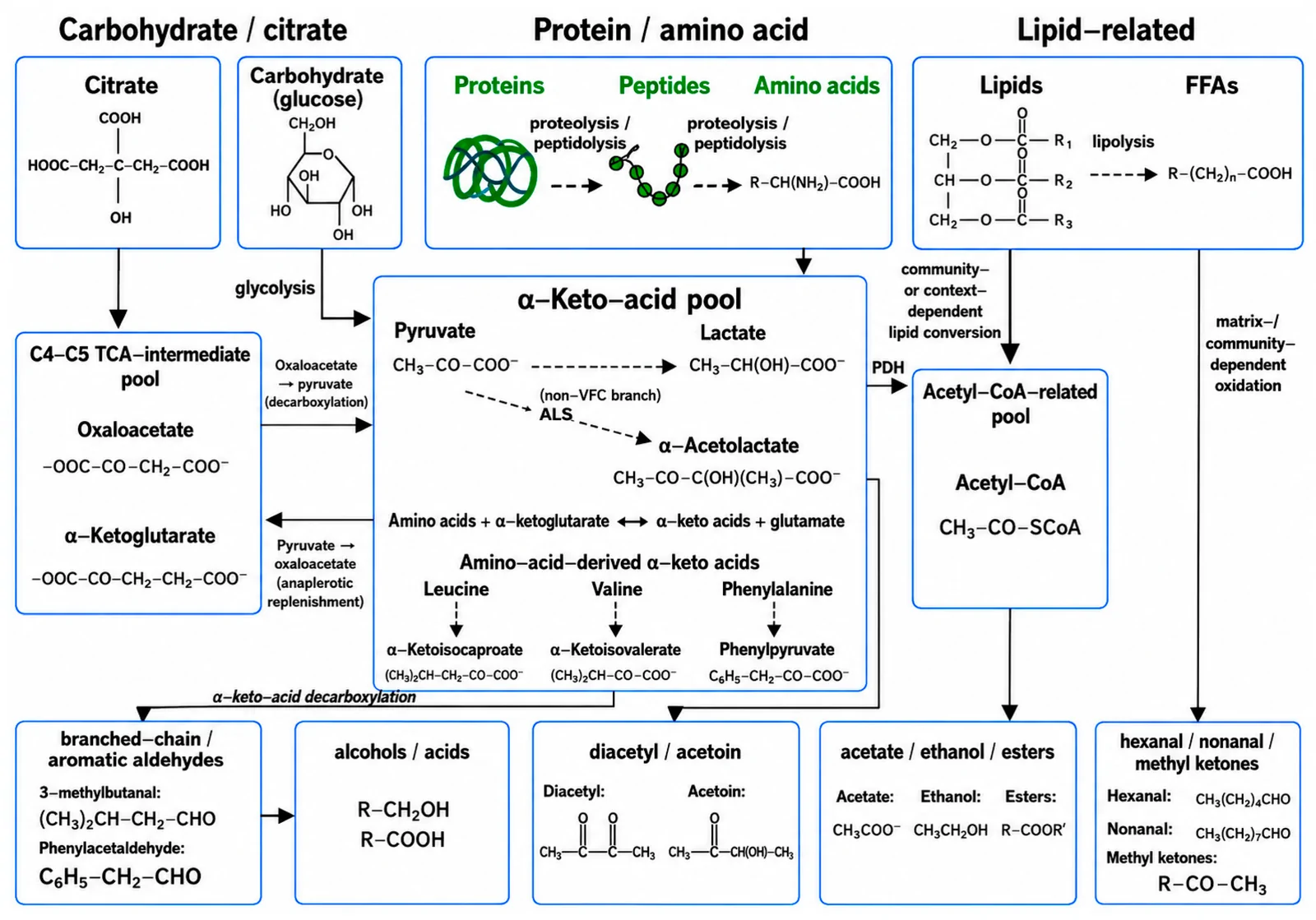
Precursor supply, convergence, and flux redistribution through three functional metabolite pools in LAB-associated VFC formation.

Biological VFC formation involves source-specific precursor generation, convergence and redistribution through shared metabolite pools, and regulation of precursor supply and branch-specific flux. Carbohydrate and citrate metabolism, protein and amino-acid metabolism, and lipid-related reactions supply the major precursors. These inputs converge through the α-keto-acid, acetyl-CoA-related, and C4–C5 TCA-intermediate pools, where genetic, intracellular, cultivation, and community-level controls alter flux partitioning and quantitative VFC formation (Figure 2).

### 2.1. Carbohydrate Metabolism

Carbohydrate metabolism is a major source of carbon skeletons entering the shared metabolite pools. In complex food matrices, fermentable sugars may be present as free sugars or may become available through matrix disruption and hydrolysis of polysaccharides. Depending on the matrix and processing conditions, acid-catalyzed hydrolysis or endogenous and microbial glycosidase/amylase activities can release oligosaccharides and monosaccharides that subsequently enter LAB carbohydrate metabolism. Glucose is converted through the Embden–Meyerhof–Parnas (EMP) pathway to pyruvate, one of the principal branch points in LAB metabolism [14]. Under strongly fermentative and oxygen-limited conditions, pyruvate is commonly reduced to lactate to regenerate NAD^+^ and sustain glycolysis [23]. In strains and conditions that permit alternative flux, pyruvate can be directed toward acetyl-CoA-related metabolism or condensed by α-acetolactate synthase (ALS) to α-acetolactate, which can subsequently yield diacetyl and acetoin-related products [20,24,25]. The balance among lactate formation, acetate/ethanol-related branches, and α-acetolactate formation depends on strain genotype, carbon-source availability, oxygen/redox conditions, and competing requirements for cofactor regeneration. This branching explains why carbohydrate metabolism affects aroma even when the dominant quantitative product remains lactic acid. Citrate provides an additional connection between carbohydrate-derived pyruvate metabolism and C4–C5 intermediates. In citrate-positive LAB, extracellular citrate can be transported into the cell [26] and cleaved by citrate lyase to oxaloacetate and acetate; oxaloacetate can then be decarboxylated to pyruvate [16,27].

Citrate utilization can increase the supply of pyruvate available for α-acetolactate formation and influence the diacetyl/acetoin balance, particularly in citrate-positive *Lactococcus* and *Leuconostoc* strains [27]. The relevant transporters, citrate-lyase components, oxaloacetate decarboxylation capacity, ALS activity, and redox state vary among strains. Citrate is treated as an upstream substrate that can replenish oxaloacetate and pyruvate-related flux, whereas the C4–C5 pool is restricted to intermediates, such as oxaloacetate and α-ketoglutarate. Representative carbonyl products provide experimentally accessible readouts of this branch. Diacetyl and acetoin reflect pyruvate/citrate allocation together with subsequent redox or oxidative conversion [20,24,27], whereas acetaldehyde and 2,3-pentanedione illustrate variation in related carbonyl outputs across dairy starter systems [28,29].

### 2.2. Protein and Amino-Acid Metabolism

Protein and amino-acid metabolism are major sources of nitrogen-containing flavor precursors and important contributors to the α-keto-acid pool [12,30]. Extracellular proteins are first hydrolyzed to peptides and free amino acids by microbial or endogenous food proteases and peptidases [30]. The released amino acids can undergo transamination, decarboxylation, deamination, and Ehrlich-type transformations. Branched-chain and aromatic amino-acid catabolism often begins with transamination in which α-ketoglutarate acts as the amino-group acceptor, forming glutamate and the corresponding α-keto acid [31,32,33]. Those α-keto acids can then be decarboxylated or otherwise converted to odor-active aldehydes, alcohols, and acids [12,13]. α-Ketoglutarate availability links the C4–C5 TCA-intermediate module to amino-acid-derived aroma formation, while the resulting α-keto acids reconnect nitrogen metabolism with the shared carbon network. Amino-acid metabolism also illustrates why flavor optimization must be evaluated together with safety and matrix context. Decarboxylation can generate metabolites, such as *γ*-aminobutyric acid (GABA) [34], while other decarboxylation routes can produce undesirable biogenic amines [35,36,37].

Some LAB can contribute to biogenic amine formation [36], whereas other strains may reduce accumulation by inhibiting amine-producing microorganisms or degrading particular amines [37]. Sulfur-containing amino acids provide precursors for methanethiol [38], hydrogen sulfide, and downstream sulfur volatiles [1,38]. The sensory effect of these products is concentration-dependent. Because precursor release in real foods may be driven by LAB, companion microorganisms, or endogenous enzymes [8,39], amino-acid-derived VFCs should be attributed to a defined strain only when the precursor-to-product conversion is experimentally resolved. 3-Methylbutanal and 3-methylbutanoic acid provide concrete downstream readouts of leucine/branched-chain-amino-acid flux through α-keto-acid intermediates [13,40,41].

### 2.3. Lipid-Related Metabolism

Lipid-related reactions are important in fermented meat, cheese, and other lipid-rich matrices, but they require particularly cautious attribution. Food lipids are hydrolyzed by microbial or endogenous lipases/esterases to free fatty acids (FFAs) [21,42], which can then undergo enzymatic oxidation, autoxidation, or, in relevant microorganisms, incomplete *β*-oxidation [6,17,43]. Unsaturated FFA oxidation can form hydroperoxides that decompose to aldehydes, ketones, alcohols, and acids [43], whereas incomplete microbial *β*-oxidation can contribute to methyl-ketone formation. Moderate lipid oxidation may enhance characteristic aroma, while excessive oxidation produces rancid notes and deterioration. In fermented meats and cheeses, staphylococci, micrococci, yeasts, and molds often have stronger lipolytic or oxidative roles than many LAB strains [8,17,39]. Lipid-derived routes are consequently treated as matrix- and community-dependent inputs rather than universal LAB pathways. Defined LAB lipase or esterase activity can support direct attribution, but the simple coexistence of LAB with increased FFAs, aldehydes, or methyl ketones is insufficient.

Lipid hydrolysis also alters the pool of acids available for subsequent biological or chemical ester formation, and changes in LAB-driven pH or ORP can indirectly change oxidation kinetics even when LAB are not the main organisms carrying out lipid conversion. This distinction between direct transformation and indirect environmental modulation is important when interpreting fermented meat and cheese studies. Lipid-derived aldehydes and methyl ketones similarly illustrate that a chemically important VFC endpoint can remain strongly community- or matrix-dependent in origin [17,21,43].

### 2.4. Three Functional Metabolite Pools as Convergence and Flux-Redistribution Nodes

Carbohydrate/citrate, protein/amino-acid, and lipid-related metabolism provide source-specific precursor inputs. These inputs are not metabolically isolated. Their carbon and nitrogen skeletons repeatedly converge at a limited set of intermediates before being redistributed toward competing VFC-forming and non-VFC branches. The three functional pools represent the network level that integrates these source pathways and provides the mechanistic basis for analyzing flux redistribution. The organizational value of the three pools rests on three practical properties rather than on the proposal of new reactions. First, they are recurrent junctions shared by more than one precursor route, allowing carbon–nitrogen coupling and cross-pathway competition to be represented without repeatedly redrawing complete carbohydrate, amino-acid, and lipid pathways. Second, they contain branch points at which strain-specific enzyme capacity, intracellular redox/cofactor balance, substrate availability, cultivation conditions, and microbial cross-feeding can redirect realized flux. Third, the same nodes can be traced to representative and measurable aroma outputs. Citrate/oxaloacetate/pyruvate allocation influences α-acetolactate availability, and the diacetyl/acetoin branch [16,20,24]; α-ketoglutarate-dependent transamination [12,44] governs formation of branched-chain-amino-acid-derived α-keto acids that can yield 3-methylbutanal and related products [13,40,41]; and acetyl-CoA-related metabolism links pyruvate-derived carbon to acetate-, ethanol-, and acyl-donor-related branches [14,15].

#### 2.4.1. C4–C5 TCA-Intermediate Pool and the Functionally Limited TCA Module

Many LAB are aerotolerant anaerobes or facultative anaerobes in which the oxidative TCA cycle is frequently incomplete or functionally limited [23,45]. The exact architecture is strain-dependent. Individual genomes or physiological states may lack, weakly express, or make limited use of one or more reactions required for a complete oxidative cycle. Nevertheless, LAB can generate or access key C4–C5 intermediates, such as oxaloacetate and α-ketoglutarate, through citrate metabolism [16], anaplerotic reactions [46], amino-acid metabolism, or other partial TCA-related routes. Citrate-positive *Lactococcus* and *Leuconostoc* strains can efficiently convert citrate-derived oxaloacetate toward pyruvate/α-acetolactate-related metabolism, whereas amino-acid-catabolizing strains may rely more strongly on glutamate dehydrogenase and aminotransferase reactions to sustain α-ketoglutarate-dependent transamination [12,16,45]. Oxaloacetate can be decarboxylated to pyruvate [47] or replenished through anaplerotic reactions [46]. The functional importance of this module lies in the supply and redistribution of C4–C5 intermediates rather than in a single universal pattern of TCA-cycle incompleteness across LAB.

The incomplete TCA cycle is treated as a potential metabolic redistribution module rather than simply as a biosynthetic limitation. Depending on strain genotype and culture conditions, this module may influence the availability of α-ketoglutarate and oxaloacetate, support amino acid transamination, and redirect acetyl-CoA-related flux toward flavor-associated branches rather than complete oxidation. This interpretation should not be generalized to all LAB without strain-level evidence. Comparative genomic, transcriptomic, metabolomic, and enzyme-activity evidence for citrate transport, citrate lyase, glutamate dehydrogenase, aminotransferases, anaplerotic enzymes, and redox enzymes is needed to support mechanistic interpretation.

#### 2.4.2. Acetyl-CoA-Related Pool

Acetyl-CoA is produced mainly from pyruvate through pyruvate dehydrogenase-related metabolism in LAB capable of this route [15]. It represents a carbon-flux convergence point connecting carbohydrate metabolism with acetate- and ethanol-related branches and with the supply of acetyl donors involved in ester metabolism. In mixed fermentations, fatty-acid-derived or amino-acid-derived carbon can also contribute to acetyl-CoA-related metabolism through companion organisms, but these routes should be treated as community-dependent unless demonstrated for a defined LAB strain. Because many LAB lack a fully oxidative TCA cycle, acetyl-CoA is often directed toward acetate, ethanol, biomass-associated reactions, or other reduced/oxidized products rather than complete oxidation. Its effect on aroma depends strongly on redox balance, substrate availability, and the organisms present. The α-acetolactate/diacetyl branch originates directly from pyruvate and is shown separately from the acetyl-CoA pool in Figure 2.

#### 2.4.3. α-Keto-Acid Pool

The α-keto-acid pool contains pyruvate and amino-acid-derived α-keto acids, such as ketoisocaproate, ketoisovalerate, and phenylpyruvate [13,14,30]. Functionally, it is the principal bridge between carbohydrate-derived carbon and amino-acid catabolism. Aminotransferases generate α-keto acids while consuming α-ketoglutarate as an amino-group acceptor [48]; the resulting α-keto acids can be enzymatically decarboxylated or oxidatively transformed to aldehydes and then reduced or oxidized to the corresponding alcohols and acids [13,31,32]. Some α-keto acids may also be reduced to hydroxy acids or transaminated back to amino acids, thereby competing with VFC-forming branches. Pyruvate has an additional role as the precursor to lactate, acetyl-CoA-related metabolism, and α-acetolactate. The pool is a functional flux module, and an increase in pool size alone should not be interpreted as enhanced aroma formation without evidence for downstream flux.

#### 2.4.4. Connections Among the Three Pools

The three pools are coupled through a limited set of mechanistically important reactions (Figure 2). Glucose-derived pyruvate can be reduced to lactate, converted through pyruvate dehydrogenase-related metabolism to acetyl-CoA, or condensed by ALS to α-acetolactate and subsequently yield diacetyl/acetoin-related products [14,15,20]. Citrate utilization generates oxaloacetate that can be decarboxylated to pyruvate [16,26,47], thereby connecting citrate uptake with the α-keto-acid network. α-Ketoglutarate, obtained through strain- and condition-dependent TCA-related, anaplerotic, or glutamate-dehydrogenase-supported reactions [44], serves as the amino-group acceptor required for many amino-acid transaminations [12,30,44]. Oxaloacetate and α-ketoglutarate connect carbon metabolism with nitrogen redistribution, while acetyl-CoA-related reactions provide an additional outlet for pyruvate-derived carbon. The practical implication is that a change in pH, oxygen, citrate, carbon/nitrogen ratio, or community composition can alter several VFC branches simultaneously by changing flux at shared nodes. These shared nodes are principal metabolic targets through which strain characteristics, intracellular regulation, and environmental conditions modify VFC formation.

The three-pool organization serves as an integration device rather than a new biochemical classification. It makes two relationships in the pathway explicit. α-Ketoglutarate-dependent transamination couples carbon and nitrogen metabolism, whereas redox-dependent partitioning controls the distribution of pyruvate and carbonyl intermediates among competing products. Because these nodes can be interrogated at gene/enzyme, metabolite/flux, and quantitative VFC levels, the same pool-centered organization also provides a bridge between mechanistic evidence and phenotype across strains and food matrices [45]. This does not mean that steady-state pool abundance is equivalent to flux; rather, the pools identify where targeted metabolite measurements, isotope tracing, enzyme assays, or controlled perturbations can be used to test whether an observed change in regulation actually propagates to aroma formation [29,45].

### 2.5. Regulation of Precursor Supply and Flux Redistribution

**Figure 3:**
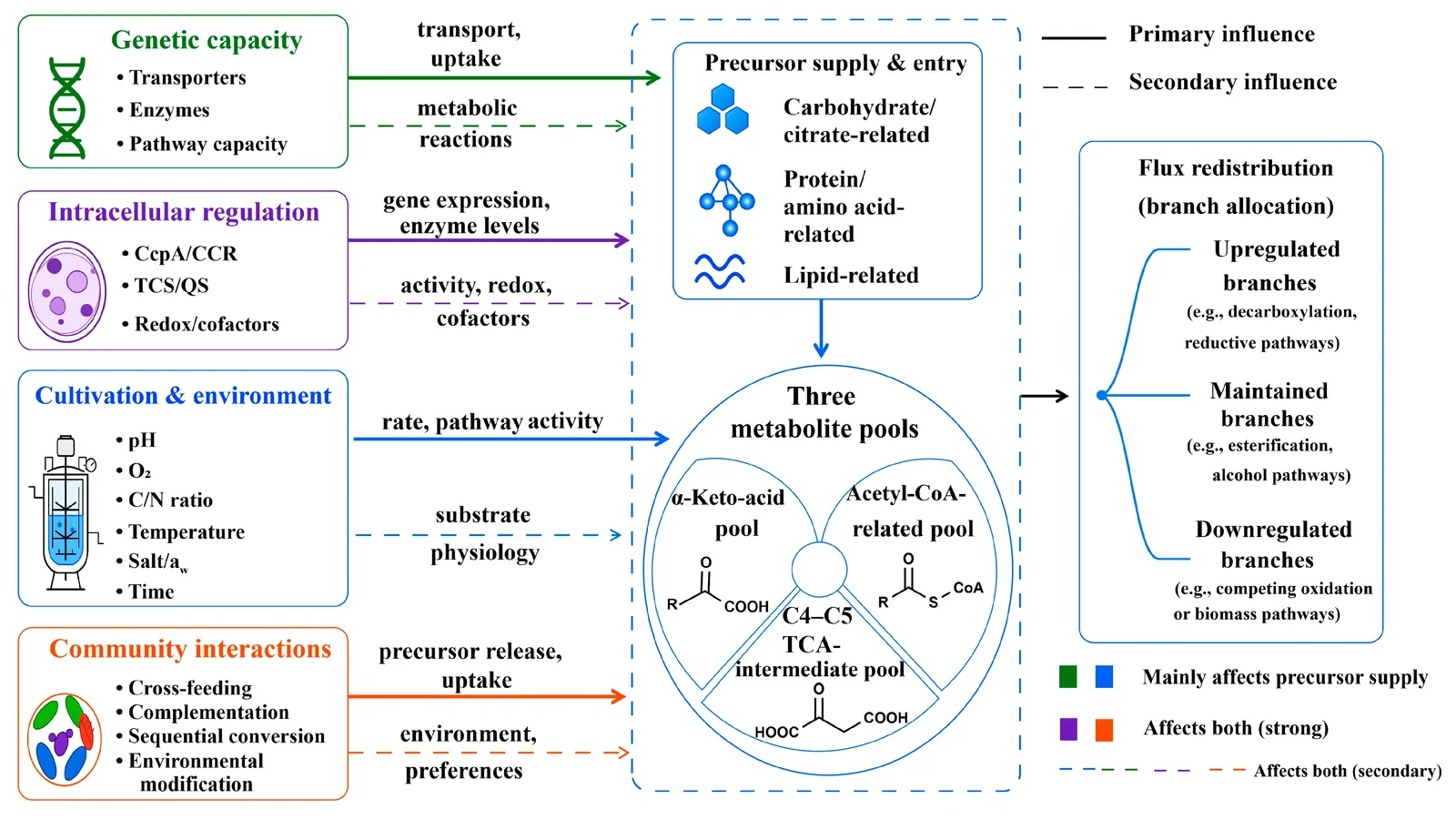
Multilevel regulation of precursor supply and flux redistribution in LAB-associated VFC formation.

Quantitative VFC output depends on both precursor supply and the partitioning of flux at shared metabolic nodes. Genetic capacity, intracellular regulation, cultivation conditions, and interspecies interactions act at both levels by changing substrate and precursor availability, transport and enzyme capacity, redox and cofactor balance, and the relative rates of competing branches. Causal attribution requires evidence that a regulatory change alters precursor availability or pool flux and that this change is propagated to quantitative VFC formation (Figure 3).

#### 2.5.1. Genetic Basis and Strain Specificity

LAB exhibit substantial strain-specific VFC-forming potential [49,50,51] because the genes, transporters, enzymes, and regulatory systems required for flavor precursor metabolism vary among strains [33,45,49] (Table 1). Carbohydrate/citrate-related functions include citrate transport [52] and cleavage (for example, *citP*/*citCL*-related modules), pyruvate dehydrogenase-related functions, and α-acetolactate synthase (als) [20,46,47]. Amino-acid-derived flavor formation depends on proteolysis/peptidolysis and on enzymes, such as branched-chain amino-acid aminotransferases (*bcaT*/*ilvE*) [31,32], glutamate dehydrogenase (*gdh*), α-keto-acid decarboxylation-related functions [33] (including *kdcA* in relevant strains), and methionine-catabolic enzymes [30,38,40]. Esterase/lipase genes can contribute to acid/alcohol or lipid-related transformations in strains in which activity has been demonstrated. These genes define metabolic capacity, but their presence alone does not establish active flux or VFC production under a specific fermentation condition. Common food-associated LAB genera discussed in flavor studies include *Lactococcus*, *Lactiplantibacillus*, *Leuconostoc*, *Pediococcus*, *Latilactobacillus*, *Lacticaseibacillus*, *Oenococcus*, *Weissella*, and *Carnobacterium*, but genus-level tendencies should be treated as hypotheses rather than deterministic traits.

Citrate-positive *Lactococcus* lactis strains, especially biovar diacetylactis, can efficiently direct citrate/pyruvate metabolism toward diacetyl and acetoin when the required transport and enzyme systems are active [20,24,52]. *Lactiplantibacillus* and *Latilactobacillus* strains differ markedly in amino-acid transamination [13,32], sulfur-amino-acid, and ester-related capacities [10,53,57]. *Leuconostoc* species can combine heterofermentative carbohydrate metabolism with citrate-related routes, whereas *Pediococcus* contributions depend strongly on strain proteolysis, salt tolerance, and matrix.

#### 2.5.2. Intracellular Regulation

The intracellular regulatory system explains how environmental signals are translated into changes in metabolic flux. Global transcriptional control [54,58], two-component and quorum-sensing systems [59], post-translational regulation, cofactor availability, and redox balance can alter substrate transport, enzyme abundance, catalytic activity, and competition among pyruvate-, amino-acid-, and citrate-related branches [60]. These layers should be viewed as control mechanisms acting on precursor supply and reaction rates rather than as independent flavor pathways. Their effects become relevant to VFC formation only when downstream metabolic and volatile changes can be demonstrated.

##### CcpA–cre-Mediated Carbon Catabolite Regulation

The carbon catabolite repression (CCR) protein CcpA is a major regulator of carbon-source utilization in many Gram-positive LAB [54,58]. Through binding to cre sites and interactions with carbon-status signals, CcpA can change the expression of carbohydrate-uptake and central-metabolic genes and, in strain-dependent contexts, alter citrate utilization, amino-acid catabolism, and other flavor-precursor branches. When rapidly metabolizable carbohydrate is abundant, flux is commonly favored toward growth and lactate-forming metabolism; under carbon limitation or when CCR is relieved, alternative pathways may become more important. The specific gene targets and direction of regulation differ among species and strains, so CcpA should be interpreted as a flux-allocation regulator rather than a universal switch that always increases or decreases a particular VFC.

##### Two-Component Systems and Quorum Sensing

Two-component systems (TCSs) and quorum-sensing (QS) mechanisms provide additional links between extracellular conditions and flavor metabolism [59,60]. In relevant strains, citrate-responsive or stress-responsive TCSs can modify expression of transport, citrate, pyruvate, or stress-response functions, while QS signals, such as autoinducing peptides (AIPs) and AI-2, can alter extracellular protease/peptidase activity [59] and other population-density-dependent functions. These mechanisms can change the rate at which peptides, amino acids, or citrate become available to the three-pool network. However, TCS and QS genes are diverse across LAB, and the presence of a signaling system should not be extrapolated to a specific VFC response unless its targets and downstream metabolic consequences have been demonstrated.

##### Post-Translational and Redox Regulation

Post-translational regulation and intracellular energy/redox state provide a faster layer of control than transcription alone [61]. Activities of the pyruvate dehydrogenase complex, glutamate dehydrogenase, alcohol/aldehyde dehydrogenases, and diacetyl/acetoin-reducing enzymes are sensitive to substrate, cofactor, oxygen, and NADH/NAD^+^ conditions [15,44,55]. The intracellular redox state can change the proportion of pyruvate reduced to lactate, the balance between aldehydes and alcohols, and the reduction of diacetyl to acetoin and 2,3-butanediol [20,55]. These relationships also explain why transcript abundance can fail to predict volatile output. The same enzyme complement can support different product ratios when cofactor balance and competing pathways change.

#### 2.5.3. Cultivation and Environmental Conditions

Cultivation and processing conditions regulate flavor through both biological and physicochemical mechanisms [62]. Carbon-source composition and the C/N ratio change substrate competition and pyruvate allocation [14]. Oxygen affects dehydrogenase/oxidase activity, cofactor regeneration, and chemical oxidation; pH changes transport, protease/peptidase [63], and decarboxylase activity, as well as the kinetics of acid-catalyzed or oxidation reactions; and ORP influences the balance between reductive and oxidative branches [55]. Salt, water activity, buffering capacity, temperature, and time modify growth, enzyme activity, substrate diffusion, volatile partitioning, and chemical reaction rates. In mixed cultures, companion microorganisms further modify these variables through oxygen consumption, acid production, proteolysis, lipolysis, and redox changes [8,17]. Environmental effects are generally range- and matrix-dependent. Acidification may promote particular protease or stress responses [60] in one strain while suppressing growth or another enzyme system in a different matrix. Oxygen can favor formation of desirable diacetyl or selected oxidation products at moderate exposure yet accelerate lipid oxidation and off-flavor formation when exposure is excessive [20,21,43].

Salt and low water activity can select for different community members and simultaneously change enzyme accessibility. Buffering capacity determines whether equivalent acid production produces the same pH trajectory. For these reasons, statements such as low pH increases flavor formation, or oxygen promotes aroma should be replaced by explicit strain, matrix, and response contexts whenever data are available. In natural fermentation, several variables change simultaneously and can generate apparently contradictory VFC responses. Oxygen and pH jointly affect pyruvate partitioning. Oxygen exposure and favorable redox conditions can increase flux toward oxidative or acetate/acetyl-CoA-related branches in some strains, whereas strongly anaerobic conditions favor lactate formation [15,23,55]. These process effects can be evaluated against defined VFC outcomes. Oxygen and redox balance can shift the diacetyl/acetoin ratio [20], carbon and nitrogen availability can alter leucine-derived 3-methylbutanal and 3-methylbutanoic acid formation [13,40,41], and excessive oxygen can increase lipid-derived hexanal, nonanal, and related oxidation products [43].

Carbon and nitrogen availability jointly influence whether amino-acid catabolism is limited by precursor supply, by α-ketoglutarate availability, or by carbon demand. Temperature and fermentation time determine both microbial succession and the duration available for enzymatic and chemical conversions. Consequently, useful process-control conclusions require multivariable or time-resolved designs rather than one-factor correlations between a cultivation parameter and a final VFC concentration. The common mechanistic denominator is flux redistribution. Genotype defines which transporters and enzymes are available; intracellular regulation and cultivation conditions alter their expression and activity; and the resulting changes in precursor supply, redox balance, and competing reaction rates redistribute carbon and nitrogen among the shared metabolite pools (Figure 3).

#### 2.5.4. Interspecies Interactions and Community-Level Flux Modification

The same precursor network extends beyond the focal LAB strain in natural and mixed fermentations. Companion organisms can alter precursor supply, remove or add intermediates, and change pH, ORP, or oxygen availability. These effects modify the same precursor network that operates within the focal LAB strain. In natural fermentations, LAB operate within ecological networks that include yeasts, staphylococci, micrococci, molds, acetic acid bacteria, and multiple LAB strains. Pure-culture fermentation may fail to reproduce the volatile profile of the corresponding natural or mixed process [64]. Four interaction mechanisms are especially relevant to VFC formation. They include metabolite or cofactor cross-feeding, functional complementation of weak or absent pathways, environmental modification through pH, ORP, or oxygen changes, and sequential conversion in which one organism supplies a precursor for another organism [8,65]. These mechanisms can change both the size of the shared precursor pools and the direction in which those pools are consumed. Interspecies metabolic complementation is evident across diverse matrices.

Yeasts can supply ethanol, amino acids, vitamins, α-ketoglutarate, or ester precursors that support LAB amino-acid catabolism and community-level ester formation. In sourdough, yeast-derived ethanol and other metabolites coexist with LAB-derived organic acids and amino-acid precursors [66], while baking provides a later chemical stage for aroma formation [54]. Cocoa fermentation involves sequential activities of yeasts, LAB, and acetic acid bacteria that reshape alcohols, organic acids, amino acids, and reducing sugars before drying and roasting [18,67]. In fermented meats, *Staphylococci* and related organisms can compensate for weak macromolecular hydrolysis or lipid conversion by LAB [17] and enhance the supply of peptides, amino acids, fatty acids, aldehydes, methyl ketones, and esters [17,53]. These examples show why community composition affects VFC formation through precursor supply as well as direct product formation [57,68]. Environmental modification is equally important. LAB acidification can suppress some microorganisms while favoring acid-tolerant community members, and LAB-associated changes in ORP can alter oxidation or reduction reactions performed by other organisms.

Conversely, yeasts can consume oxygen, release growth factors, and produce ethanol; acetic acid bacteria can oxidize alcohol-derived substrates; molds can change matrix structure and release peptides or fatty acids. Such interactions mean that a companion organism can change a LAB-associated VFC profile even without transferring a measurable flavor precursor, simply by changing the conditions under which LAB enzymes operate. Composite LAB communities can also remodel the network through strain-to-strain cross-feeding [56,69]. A citrate-positive or precursor-supplying strain may enrich pyruvate, oxaloacetate-related intermediates, amino acids, peptides, or vitamins that are subsequently used by a strain with stronger amino-acid-catabolic or ester-related capacity. Conversely, a strain with strong proteolysis can release amino acids that become substrates for another strain. Most published co-fermentation studies, however, report differences in final volatile profiles without resolving precursor donors and product-forming strains. When individual contributions cannot be separated, volatile changes are described as LAB-associated system-level outcomes. Defined synthetic communities, strain-resolved transcriptomics/metabolomics, isotope-labeled substrates, and time-resolved volatile profiling are preferred for distinguishing cross-feeding from parallel metabolism and for testing whether a companion organism truly changes flux through the proposed pools.

### 2.6. Mechanistic Evidence for Biological VFC Formation

The preceding sections describe how precursor pathways converge at shared metabolic nodes and how multilevel regulation influences VFC formation. Mechanistic interpretation therefore requires linking metabolic potential with functional activity, precursor transformation, flux redistribution, and quantitative VFC production. Because individual analytical approaches address different biological questions, stronger conclusions require convergence of molecular, metabolic, flux, chemical, and sensory evidence.

#### 2.6.1. Molecular, Metabolic, and Flux Evidence

Genomic, transcriptomic, proteomic, and enzyme-level analyses provide evidence for the metabolic potential and functional capacity involved in precursor generation and VFC formation. Relevant functions include citrate utilization, pyruvate conversion, amino-acid catabolism, α-keto-acid metabolism, and strain-specific esterase or lipase activities [39,49]. However, gene presence or expression alone cannot demonstrate realized metabolic flux, because substrate availability, cofactors, redox state, transport, and competition among pathways can alter metabolic outcomes [43,48,49].

Metabolite measurements provide a closer link between functional activity and VFC formation. Targeted analysis of precursor depletion, intermediate changes, and downstream products can evaluate whether the proposed precursor–product relationship occurs. Untargeted metabolomics can identify candidate metabolic nodes and coordinated responses [70], whereas targeted and time-resolved measurements provide stronger evidence for metabolic sequence and flux behavior. Stable-isotope tracing provides further support by directly tracking precursor contribution to downstream metabolites and VFCs.

These evidence layers are complementary rather than interchangeable. Quantitative VFC analysis defines the chemical outcome [29], while GC–olfactometry, OAV/ROAV analysis, sensory evaluation, and aroma recombination or omission experiments determine whether these changes contribute to aroma perception [3]. Network-level regulation is most convincingly supported when controlled perturbations, functional measurements, flux-related evidence, and VFC outcomes converge. Sensory evidence is additionally required when conclusions extend from chemical formation to desired flavor.

#### 2.6.2. Causal Attribution in Mixed Fermentations

Causal attribution becomes more complex in mixed fermentations because precursor release, intermediate conversion, and final VFC formation may involve different microorganisms. Community profiling combined with metabolomics, transcriptomics, or volatilomics can identify candidate microorganism–metabolite relationships [51,52,61,66], but such associations alone cannot demonstrate direct biosynthesis by a defined LAB strain.

Companion microorganisms may provide precursors, cofactors, or environmental changes that modify LAB-associated VFC formation. For example, proteolytic microorganisms can release peptides and amino acids, yeasts can provide ethanol, vitamins, amino acids, or α-ketoglutarate, and other microorganisms may contribute lipid-derived precursors [29,51,52,53]. Therefore, direct attribution to a LAB strain requires evidence linking metabolic capacity, precursor conversion, and quantitative VFC formation. Without strain-resolved functional validation, isotope tracing, or controlled community experiments, volatile profiles should be interpreted as LAB-associated system-level outcomes rather than direct LAB-derived biosynthesis.

## 3. Biological and Non-Enzymatic Processes in VFC Formation

**Figure 4:**
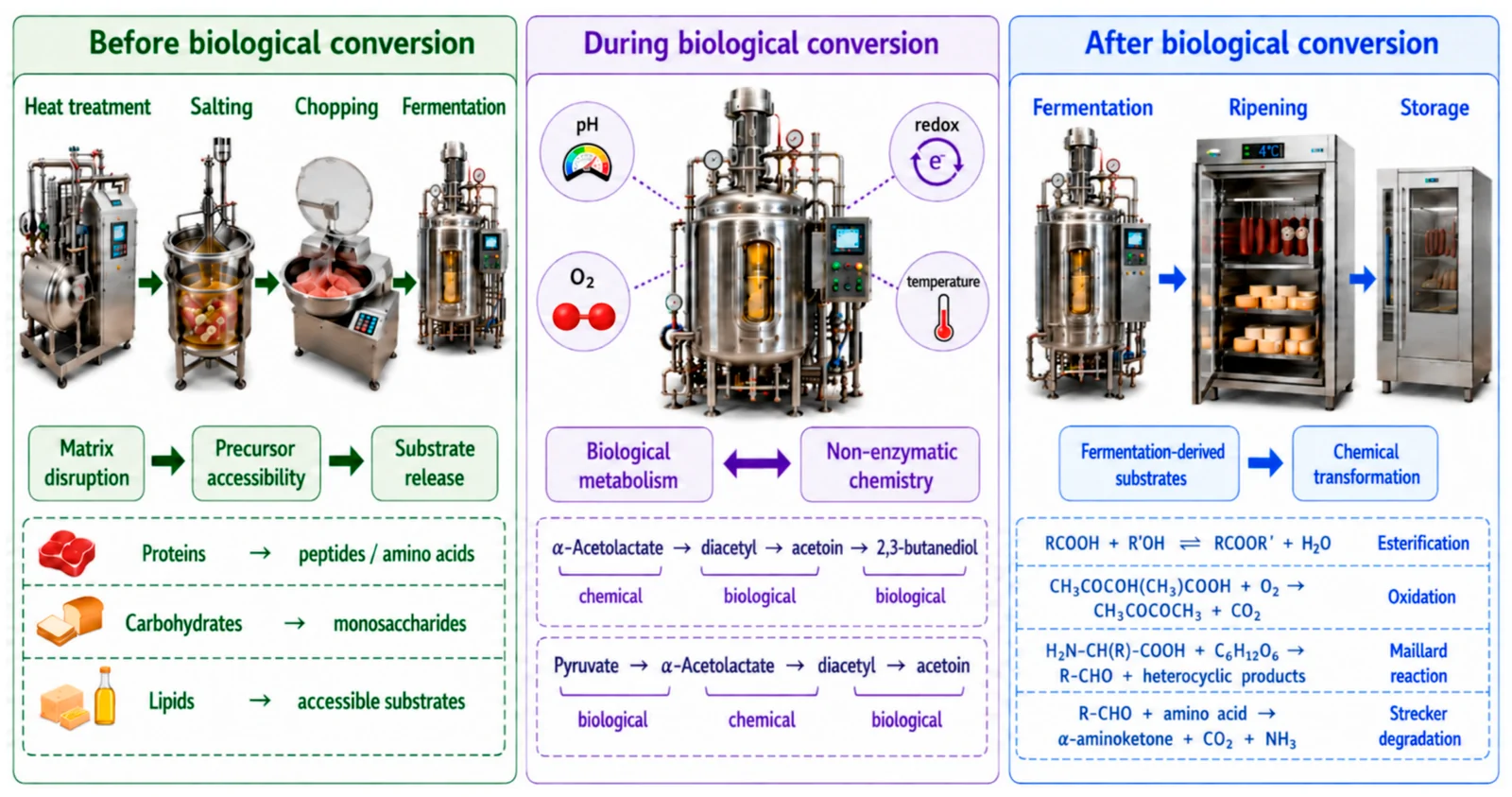
Temporal configurations of biological and non-enzymatic processes in LAB-associated VFC formation.

Non-enzymatic chemistry contributes to VFC formation in many fermented foods, but the extent to which these reactions depend on biological metabolism is matrix- and process-specific. The term biological and non-enzymatic coupling is reserved for cases supported by a temporal, substrate, or physicochemical relationship between biological and chemical steps. Three configurations are distinguished for mechanistic analysis. physicochemical modification that changes precursor availability before microbial conversion, chemical transformation of fermentation-derived substrates after biological activity, and concurrent biological and chemical reactions during fermentation, ripening, storage, or processing. Where chemical conversion is documented without such dependence, it should be interpreted as an independent non-enzymatic contribution rather than as evidence of coupling. Mechanistic attribution consequently requires separate identification of precursor origin, biological conversion, chemical conversion, and the process conditions under which each step occurs (Figure 4).

### 3.1. Physicochemical Precursor Release Before Fermentation

Physical and chemical pretreatments, such as heating, acidification or acidolysis, salting, chopping, pulsed or other structure-disrupting treatments, can release small molecules or increase the accessibility of macromolecular substrates before fermentation [62,68]. Protein unfolding and tissue disruption can increase exposure to proteases; disruption of plant or cereal matrices can improve access to carbohydrate substrates, while acid-catalyzed hydrolysis under appropriate conditions may further depolymerize polysaccharides and increase the availability of fermentable oligosaccharides or monosaccharides; lipolysis or structural changes can increase the availability of FFAs. These effects alter what enters the subsequent microbial network. Enzymatic hydrolysis can serve a similar precursor-release function, but because it is itself biologically catalyzed, it is treated here as a biological pretreatment mechanism rather than as a non-enzymatic reaction. In fermented sausages, salting, chopping, and ripening facilitate release of peptides, amino acids, and FFAs [21,70], and LAB–staphylococcal combinations can subsequently convert these materials into aldehydes, ketones, acids, and esters [53]. In plant and cereal matrices, heating, acidification, or structural disruption can expose reducing sugars, proteins, and organic acids that later enter LAB and community metabolism.

The benefit of pretreatment is conditional. Increased precursor accessibility can enhance desirable pathways, while excessive heat, oxidation, acid treatment, or mechanical disruption can destroy precursors, inhibit microorganisms, or generate substrates for off-flavor reactions. The appropriate intensity must be optimized for the matrix and starter community rather than generalized across fermented foods.

### 3.2. Chemical Modification After Fermentation

Post-fermentation chemical modification can alter VFC composition without additional microbial synthesis; representative routes include α-acetolactate oxidation, sulfur-compound oxidation, and ester formation from accumulated acids and alcohols.

#### 3.2.1. Esterification Reaction

LAB fermentation leaves a chemically reactive product matrix containing organic acids, alcohols, α-keto acids, amino acids, reducing sugars, sulfur-containing precursors, and lipid-derived substrates [14]. Ester formation requires an explicit mechanistic distinction between microbial catalysis and chemical esterification. LAB can supply lactic and acetic acids, and LAB or companion microorganisms can supply ethanol, 3-methylbutanol [71], phenethyl alcohol, and other alcohols [64,71]. Microbial esterases or acyltransferase-related activities can catalyze ester formation in relevant organisms, while acid-catalyzed esterification can occur chemically when acids and alcohols coexist under favorable pH, water activity, temperature, and storage conditions [22]. Ethyl acetate, isoamyl acetate, and phenethyl acetate can arise from routes that are chemically similar at the product level but biologically different at the source level. Claims of direct LAB ester production require evidence that distinguishes these possibilities.

#### 3.2.2. Non-Enzymatic Oxidation

Non-enzymatic oxidation constitutes a major post-fermentation transformation route. α-Acetolactate generated biologically from pyruvate can undergo oxidative decarboxylation to diacetyl during fermentation, ripening, or storage [20]. Methanethiol and related sulfur precursors can be oxidized to dimethyl disulfide or dimethyl trisulfide [10,62]. FFAs released through lipolysis can undergo autoxidation to hydroperoxides and downstream aldehydes, ketones, alcohols, and acids [21,43]. Hexanal and nonanal are representative lipid-oxidation products, but their sensory value depends on concentration and matrix. Moderate oxidation may provide characteristic buttery, fatty, green, or mature notes, while excessive oxygen exposure accelerates rancidity and other off-flavors. Oxygen management should be treated as a balance between desired oxidation and deterioration.

#### 3.2.3. Maillard and Strecker Reactions

Fermentation can increase the availability of substrates for thermally induced aroma chemistry [19,72]. Free amino acids, peptides, and reducing sugars accumulated during sourdough, cocoa, meat, cereal, or other fermentations become reactants for Maillard chemistry during baking, roasting, or cooking [18,19,66]. Strecker degradation of branched-chain or aromatic amino acids with reactive carbonyl compounds can form aldehydes, such as 3-methylbutanal and phenylacetaldehyde, while broader Maillard chemistry generates furans, pyrroles, pyrazines, and other roasted or caramelized odorants. This distinction defines an important attribution boundary. Fermentation may increase precursor availability and alter pH or redox conditions, whereas the final odorants can be generated predominantly during subsequent heating rather than by LAB metabolism. Excessive thermal conversion can also increase burnt notes or process-related contaminants [73], so flavor enhancement and safety must be evaluated together.

### 3.3. Overlapping Biological and Non-Enzymatic Reactions

#### 3.3.1. pH-Mediated Coupling

In natural fermentations, biological metabolism and chemical reactions often overlap rather than occurring in discrete stages. pH is a central coupling variable because LAB acidification simultaneously changes microbial succession [22], transporter and enzyme activity, substrate ionization, and the kinetics of acid-sensitive chemical reactions. In fermented vegetables, for example, LAB-driven acidification can influence peptidase activity, sulfur-precursor availability, and subsequent oxidation processes [10,63,74]. A staged pH trajectory can have different consequences from a constant endpoint pH. Early conditions may favor precursor release and microbial conversion, while later acidification stabilizes the product and suppresses spoilage. The optimal trajectory is matrix- and starter-dependent.

#### 3.3.2. Bidirectional Substrate–Product Coupling in the Diacetyl–Acetoin System

The diacetyl–acetoin system exemplifies temporally overlapping biological and chemical conversion. Two molecules of pyruvate are condensed enzymatically by ALS to α-acetolactate. α-Acetolactate can undergo oxidative decarboxylation to diacetyl, while diacetyl can be biologically reduced to acetoin and acetoin further reduced to 2,3-butanediol [20,24]. Oxygen exposure, redox state, pH, and the activity of reductases determine the ratio between a strong buttery odorant and its less intense downstream reduction products. The final diacetyl concentration cannot be interpreted solely from ALS gene expression or α-acetolactate formation because chemical oxidation and biological reduction occur in the same process window.

#### 3.3.3. ORP-Mediated Oxidation–Reduction Balance

ORP regulates the balance between oxidative and reductive transformations in overlapping biological and chemical processes. Moderate oxidative conditions can favor formation of selected carbonyls, sulfur oxidation products, and α-acetolactate-derived diacetyl, whereas a strongly reducing environment favors conversion of aldehydes to alcohols and diacetyl to acetoin/2,3-butanediol [20,43]. Excessive oxidation accelerates FFA autoxidation and rancid-aldehyde formation [21,43] and can intensify sulfur notes beyond desirable levels. LAB may modify ORP through metabolism and strain-dependent antioxidant or redox systems [75,76], while yeasts and other community members also consume oxygen or supply reducing/oxidizing metabolites. Process control requires coordinated management of oxygen transfer, ORP, pH, and ripening/storage time rather than optimization of any one variable in isolation. Process regulation should be assessed primarily by defined quantitative VFC outcomes; odor-activity or sensory endpoints are additionally required only when an intervention is claimed to improve characteristic aroma. Across these temporal configurations, mechanistic attribution requires resolution of precursor source, reaction timing, biological or chemical conversion step, and quantitative VFC outcome. The three functional pools are restricted to the organization of biological metabolism; non-enzymatic steps are described independently unless direct precursor or process continuity is supported.

## 4. The Formation of Key Aroma Components During LAB Fermentation

**Figure 5:**
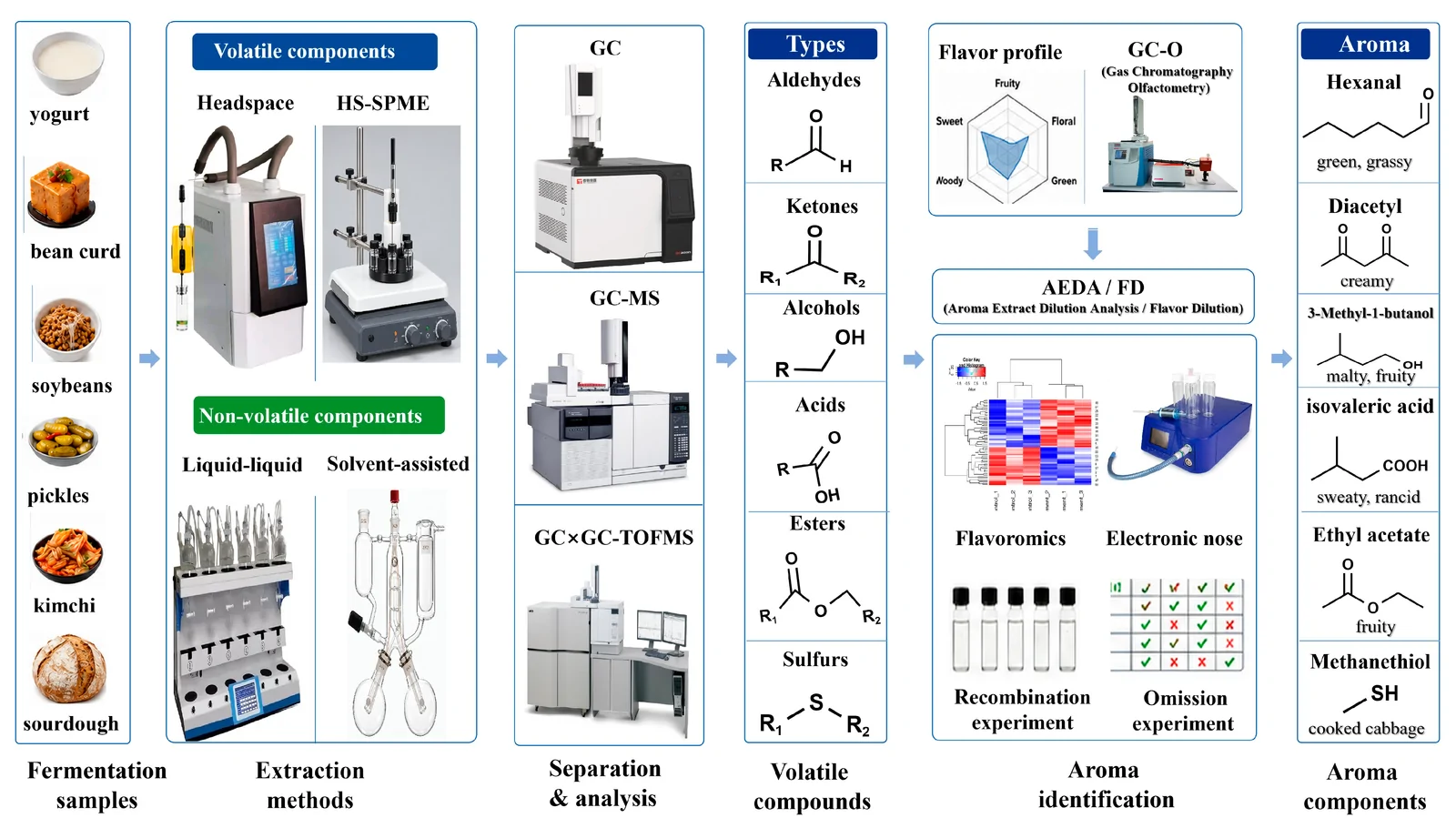
Workflow for identification and validation of key aroma-active compounds in LAB-fermented foods.

The biological and biological–non-enzymatic processes described above ultimately generate a complex volatile phenotype, but only a subset of the resulting volatile flavor compounds (VFCs) contributes substantially to the characteristic aroma of fermented foods. Interpretation of LAB-associated flavor formation should therefore proceed from comprehensive characterization of fermentation-associated volatilomes to identification of aroma-active compounds and integration of these sensory-relevant endpoints with the formation networks established in the preceding sections (Figure 5). This distinction is important because chemical abundance, aroma activity, and formation origin represent related but non-equivalent properties of a VFC.

### 4.1. Volatile Profiling of Fermentation-Associated VFCs

LAB-containing fermentations generate chemically diverse volatile profiles, including aldehydes, ketones, alcohols, organic acids, esters, sulfur-containing compounds, lipid-derived volatiles, and other matrix-dependent products [1,2,11]. Their composition varies with LAB strain, companion microorganisms, precursor availability, fermentation stage, food matrix, and processing conditions (Table 2). Comprehensive volatile profiling, therefore, provides the chemical output against which the biological and biological–non-enzymatic mechanisms described above can be evaluated.

HS-SPME–GC–MS approaches are widely used for comparative and quantitative profiling of fermentation-associated VFCs because they provide broad volatile coverage with relatively simple sample preparation. GC–IMS can additionally support rapid comparison of volatile fingerprints among strains, fermentation stages, or processing conditions when compound identification and quantitative interpretation are appropriately controlled [3,29].

These analytical approaches are complementary rather than interchangeable. Relative peak abundance alone should not be interpreted as a direct measure of sensory contribution or biosynthetic importance because odor thresholds, matrix retention, release behavior, and compound interactions strongly influence aroma perception [3,4]. The resulting volatile profile should therefore be regarded primarily as the chemical phenotype of fermentation. Detection of diacetyl, 3-methylbutanal, phenylacetaldehyde, esters, sulfur compounds, or lipid-derived aldehydes demonstrates their presence in the final volatile mixture but does not by itself establish whether they were produced directly by LAB, generated by companion microorganisms, or formed through subsequent chemical transformation.

The formation origin of individual VFCs requires integration of precursor metabolism, microbial composition, matrix characteristics, and processing conditions. For example, diacetyl and acetoin reflect pyruvate/citrate-related metabolism and subsequent redox-dependent conversion [20,24,27], whereas amino-acid-derived aldehydes, such as 3-methylbutanal, are associated with α-keto-acid pathways [40,41]. Lipid-derived aldehydes and sulfur-containing compounds require additional consideration of matrix composition, microbial interactions, oxidation conditions, and processing history [1,10,38,65].

### 4.2. Identification and Validation of Key Aroma-Active VFCs

Comprehensive volatile profiling defines the chemical space generated during fermentation, but key aroma-active compounds cannot be identified solely according to abundance. Molecular sensory approaches are therefore required to determine which VFCs contribute directly to characteristic aroma. GC–olfactometry (GC–O), aroma extract dilution analysis (AEDA), odor activity evaluation, and sensory validation approaches provide complementary evidence for identifying aroma-relevant compounds [4,28].

GC–O and AEDA identify odor-active regions within complex volatile extracts, while odor activity values (OAVs) or related approaches help prioritize compounds with concentrations exceeding their odor thresholds. However, these indicators remain dependent on matrix characteristics, volatile release, and interactions among coexisting odorants. Therefore, odor activity evidence should be interpreted together with quantitative analysis and formation-route information rather than as independent proof of aroma contribution [3,4].

Aroma recombination and omission/addition experiments provide stronger validation of the contribution of selected compounds. Recombination tests evaluate whether a defined mixture of route-linked odorants can reproduce the characteristic aroma of the original product, whereas omission or addition experiments determine whether removal or introduction of specific compounds causes perceptible changes [4,28]. These approaches are particularly important because characteristic aroma is an emergent property of complex mixtures rather than a simple consequence of individual compound concentrations.

Accordingly, the identification of a key aroma-active VFC is strongest when chromatographic–olfactory detection, accurate quantification, odor-activity assessment, and sensory validation converge. However, sensory confirmation does not establish formation origin. A compound may be demonstrated to contribute strongly to characteristic aroma while its producing microorganism or chemical formation route remains unresolved.

### 4.3. Integration of Key Aroma-Active VFCs with Formation Networks

Once key aroma-active VFCs have been identified, their significance can be interpreted within the precursor–pool–flux and biological–non-enzymatic frameworks developed above. This integration should focus on linking aroma-relevant endpoints with previously defined formation modules rather than repeating complete metabolic pathways (Table 3).

Diacetyl and acetoin represent characteristic aroma endpoints associated with pyruvate/citrate-related carbon redistribution and subsequent biological or chemical conversion processes [20,24,27]. Amino-acid-derived aldehydes, such as 3-methylbutanal and phenylacetaldehyde, represent aroma-active outputs associated with α-keto-acid metabolism and amino-acid catabolism [40,41]. Esters reflect the combined availability of alcohol and acid precursors and may involve enzymatic esterification as well as subsequent chemical reactions depending on the fermentation system [22,64,72]. Sulfur-containing compounds and lipid-derived aldehydes or ketones often depend more strongly on companion microorganisms, matrix composition, oxidation conditions, and non-enzymatic chemistry [1,10,38].

The strength of source attribution differs among aroma-active endpoints. Some compounds, such as diacetyl and specific amino-acid-derived aldehydes, can be linked relatively directly to defined LAB metabolic pathways under controlled conditions. In contrast, esters, sulfur compounds, and lipid-derived odorants frequently represent system-level outcomes influenced by microbial communities and food matrices. Their sensory importance may therefore be well established even when direct LAB biosynthesis has not been demonstrated.

Sensory validation thus narrows the broader VFC formation network to those biological branches and biological–non-enzymatic interfaces that generate aroma-relevant endpoints. Comprehensive volatile profiling identifies what is formed, molecular sensory analysis determines which products contribute to characteristic aroma, and formation-network integration establishes where these key odorants fit within the mechanistic framework. In this way, characteristic aroma becomes the sensory consequence of LAB-associated VFC formation rather than an independent analytical endpoint.

## 5. Conclusions and Perspectives

LAB-associated VFC formation is principally controlled by biological metabolism, in which carbohydrate/citrate-, amino-acid-, and lipid-related pathways are connected through the α-keto-acid, acetyl-CoA-related, and C4–C5 TCA-intermediate pools. These pools are operational convergence and redistribution nodes rather than independent pathways or new biochemical classes; their principal value is to organize established pathway-specific evidence at shared points where precursor availability and branch competition can be experimentally examined. Strain genotype defines metabolic capacity, whereas intracellular regulation, cultivation conditions, and community interactions determine realized flux through these nodes. Robust causal interpretation requires convergence of functional activity, precursor or pool measurements, flux-resolved evidence where feasible, and quantitative VFC outcomes under defined strain, community, and matrix conditions. Non-enzymatic chemistry constitutes an additional mechanism of VFC formation whose relationship with biological metabolism depends on processing context. Physicochemical treatments can alter precursor accessibility before fermentation, fermentation-derived substrates can undergo chemical transformation after biological activity, and biological and chemical reactions can also proceed concurrently.

α-Acetolactate oxidation to diacetyl, ester formation from fermentation-derived acids and alcohols, and Maillard/Strecker conversion of accumulated amino acids and reducing sugars illustrate cases in which microbial activity changes the substrates available to subsequent chemical reactions. Chemical routes that lack demonstrable precursor or process dependence on microbial metabolism require independent attribution. Volatile profiling defines the chemical phenotype produced by biological and non-enzymatic mechanisms, while flavoromics can prioritize discriminant VFCs or VFC combinations for route-specific interpretation. When conclusions extend to characteristic or desired aroma, these candidates should be evaluated by GC–olfactometry/AEDA, OAV/ROAV, molecular sensory analysis, and, where feasible, recombination/omission testing. Such evidence validates perceptual contribution but does not substitute for source-resolved mechanistic evidence.

Future studies should prioritize strain-resolved functional validation, targeted measurement of the three metabolite pools, stable-isotope or other flux-resolved approaches, time-resolved community experiments, and kinetic analysis of biological and non-enzymatic transformations. Combining formation-level measurements with volatilomics/flavoromics and mechanism-informed sensory validation in the same time-resolved design would permit direct assessment of whether redirected precursor flux produces the intended aroma-active outcome while limiting off-flavor and safety risks.

## Figures and Tables

**Table 1:** Core flavor-related genes and representative VFCs of food-associated LAB.

LAB Genus	Main Genes/Modules	Key Enzymes/Pool-Control Node	Representative VFCs	Flavor Notes	References
*Pediococcus*	*ldh*, *pep*, *bcaT*/*ilvE*, est	LDH, peptidases, BCAT LDH, Pool/node emphasized here: α-Keto-acid precursor supply; strain-dependent ester branch	Lactate, branched aldehydes, esters	Sour, mild fruity	[7,14]
*Weissella*	Heterolactic genes, *ldh*, *pep*, *bcaT*, est	LDH, peptidases, esterase LDH, Pool/node emphasized here: Pyruvate/α-keto-acid node; ester-related branch	Lactate, acetate, ethanol, esters	Sour, fruity, fermented	[7,14]
*Latilactobacillus*	*ldh*, *pep*, *bcaT*, *kdcA*, est	Peptidases, BCAT, decarboxylase Pool/node emphasized here: α-Keto-acid pool (BCAA-derived flux)	3-methylbutanal, alcohols, acids, esters	Malty, meaty, fermented	[9,13,32]
*Carnobacterium*	*citP*, *citCL*, *oad*, *pep*, est	Citrate lyase, OAD, peptidases Pool/node emphasized here: C4–C5 citrate link and α-keto-acid precursor supply	Acetate, diacetyl precursors, AA-derived volatiles	Acidic, dairy-like	[30,47]
*Oenococcus*	*mleA*, *mleP*, *citP*, *citCL*	Malolactic enzyme, citrate lyase Pool/node emphasized here: C4–C5 citrate link–pyruvate/α-keto-acid node	Lactate, diacetyl, acetoin, acetate	Winey, buttery	[16,46,51]
*Lactococcus*	*citP*, *citCL*, *als*, *pdh*, *bcaT*/*ilvE*	Citrate lyase, ALS, BCAT Pool/node emphasized here: C4–C5 citrate link–pyruvate/α-keto-acid node	Diacetyl, acetoin, acetaldehyde, 3-methylbutanal	Buttery, dairy, malty	[20,31,40,52]
*Lactiplantibacillus*	*pts*, *ldh*, *citP*, *bcaT*, *gdh*, *mgl*, est	LDH, BCAT, GDH, esterase LDH, BCAT, GDH, Pool/node emphasized here: α-Keto-acid/C4–C5 coupling; ester-related acetyl-CoA branch	Lactate, acetate, aldehydes, alcohols, esters	Sour, fruity, fermented	[10,26,53]
*Lacticaseibacillus*	*lac*, *lacZ*, *ldh*, *ccpA*, *pep*, est	*β*-Galactosidase, LDH, peptidases *β*- Pool/node emphasized here: Pyruvate/α-keto-acid node; ester-related branch	Lactate, acetaldehyde, acetoin, esters	Dairy, acidic, fruity	[14,50,54]
*Leuconostoc*	Heterolactic genes, *citrate*, est	XPK, citrate enzymes, esterase XPK, Pool/node emphasized here: C4–C5 citrate link–pyruvate/α-keto-acid node	Diacetyl, acetoin, acetate, ethanol, esters	Buttery, acidic, fruity	[14,55]
*Lactobacillus s.*	*lacZ*, *lacS*, *ldh*, *pep*, *bcaT*, est	*β*-Galactosidase, peptidases, BCAT *β*- Pool/node emphasized here: Pyruvate/α-keto-acid and amino-acid-derived α-keto-acid nodes	Lactate, acetaldehyde, diacetyl, aldehydes	Dairy, sour, roasted	[1,14,56]

**Table 2:** Major classes, representative compounds, formation routes, and sensory interpretation of LAB-associated VFCs.

VFC Class VFC	Representative Compounds	Main Routes Emphasized in This Review	Typical Sensory Contribution	Attribution Caution	References
Carbonyls and ketones	Diacetyl; acetoin; acetaldehyde; 3-methylbutanal; phenylacetaldehyde; methyl ketones; hexanal/nonanal	Pyruvate/citrate metabolism; amino-acid catabolism; lipid oxidation	Buttery, malty, floral, green/fatty, matrix dependent	Lipid-derived and some mixed-fermentation products often require community-level attribution	[13,20,43]
Alcohols	Ethanol; 3-methylbutanol; phenethyl alcohol	Heterolactic/carbohydrate metabolism; reduction of amino-acid-derived aldehydes; co-fermentation	Fermented, fruity, floral	Yeasts can be major contributors in mixed fermentations	[13,64,71]
Organic acids	Acetic acid; branched-chain acids	Carbohydrate metabolism; amino-acid catabolism	Acidic, fermented; also ester precursors	Concentration and buffering strongly affect sensory effect	[13,14,71]
Esters	Ethyl acetate; isoamyl acetate; phenethyl acetate	Biological ester formation and/or acid-catalyzed esterification from alcohols and organic acids	Fruity, floral	Source should be separated between LAB, yeasts/other microbes, and chemical esterification	[22,64,71]
Sulfur volatiles	Methanethiol; dimethyl disulfide; dimethyl trisulfide	Sulfur-amino-acid catabolism followed by biological or chemical oxidation	Sulfurous, cooked, savory; off-note at excessive levels	Final products can arise after LAB precursor formation through oxidation	[1,10,38]

**Table 3:** Representative aroma-active compounds, formation routes, and attribution constraints in LAB-associated fermented foods.

Characteristic Odorant(s)	Representative LAB/Matrix Context	Formation Route Emphasized in This Review	Typical Sensory Contribution	Attribution/Interpretive Note	References
Diacetyl/acetoin	Citrate-positive *Lactococcus*/*Leuconostoc* and other dairy LAB; fermented dairy	Citrate/pyruvate–α-acetolactate–diacetyl; biological reduction–acetoin	Buttery, creamy, dairy-like	Combines biological precursor formation with redox/oxygen-dependent chemical conversion and biological reduction	[20,27,28]
Acetaldehyde/2,3-pentanedione	Yogurt and fermented dairy starter systems	Pyruvate/carbohydrate-related carbonyl formation; strain- and process-dependent	Fresh/yogurt-like; buttery/caramel-like	LAB-associated endpoint unless the producing strain and route are resolved	[1,28,29]
3-Methylbutanal/3-methylbutanoic acid/3-	*Lactococcus lactis* and *Latilactobacillus sakei* models; fermented meat/cheese	Leucine–α-ketoisocaproate–decarboxylation and subsequent redox conversion	Malty, nutty, ripened; cheesy/pungent at high levels	Direct strain-level conversion is supported in defined systems; sensory effect remains concentration dependent	[13,40,41]
Phenylacetaldehyde/phenethyl alcohol	LAB-containing fermented foods and co-fermentations	Aromatic amino-acid transamination/decarboxylation followed by reduction/oxidation	Floral, honey-like, rose-like	Often system-level; direct LAB contribution depends on strain and companion organisms	[12,64,71]
Ethyl acetate/isoamyl acetate/phenethyl acetate	LAB–yeast co-fermentations and ripened/stored fermented foods	Alcohol + acid -microbial esterification and/or acid-catalyzed esterification	Fruity, floral	Attribution requires separation of LAB, yeast/other microbial, and chemical contributions	[22,64,71]
Methanethiol/DMDS/DMTS	Cheese-ripening and fermented vegetable systems	Methionine catabolism -methanethiol -biological/chemical oxidation	Sulfurous, cooked, savory; off-note when excessive	LAB may supply sulfur precursors, whereas downstream oxidation can be biological or chemical	[1,10,38]
Hexanal/nonanal/methyl ketones	Fermented meat, cheese, and other lipid-rich matrices	FFA release followed by enzymatic oxidation, autoxidation, or context-dependent *β*-oxidation/FFA	Green, fatty, mature; rancid when excessive	Strongly matrix- and community-dependent; generally not assigned to LAB alone	[17,21,43]

## Data Availability

No new data were created or analyzed in this review.
